# The Adapted Italian Version of the Baller Identity Measurement Scale to Evaluate the Student-Athletes’ Identity in Relation to Gender, Age, Type of Sport, and Competition Level

**DOI:** 10.1371/journal.pone.0169278

**Published:** 2017-01-05

**Authors:** Corrado Lupo, Cristina Onesta Mosso, Flavia Guidotti, Giovanni Cugliari, Luisa Pizzigalli, Alberto Rainoldi

**Affiliations:** 1 Motor Science Research Center, School of Exercise & Sport Sciences (SUISM), Department of Medical Sciences, University of Torino, Turin, Italy; 2 Department of Psychology, University of Torino, Turin, Italy; 3 Division of Human Movement and Sport Sciences, Department of Movement, Human and Health Sciences, University of Rome Foro Italico, Rome, Italy; 4 Department of Medical Sciences, University of Torino, Turin, Italy; Universidade de Tras-os-Montes e Alto Douro, PORTUGAL

## Abstract

The purpose of this paper is twofold: to validate the properties of the Italian version of the Baller Identity Measurement Scale (i.e., BIMS-IT), a self-report questionnaire based on the athletic and academic identities; and to investigate differences in psychosocial factors such as gender, age, type of sport, and competition level. The dimensionality of the BIMS-IT was explored by means of the exploratory factor analysis, considering the scale’s internal consistency too (Confirmatory Factor Analysis). Results related to exploratory and confirmatory factor analysis supported a model of measurement composed of two correlated factors: the athletic and academic identities and affectivity related to identities. For both factors, differences emerged between age, and competition level sub groups. In particular, higher identity scores emerged for ≤ 24 years old student-athletes with respect to their age counterparts. National sub-elite student-athletes reported lower identity values than those of national elite and international levels. Results suggest that the Italian version of the BIMS-IT is psychometrically robust and could be adopted for empirical uses. The higher identity scores reported by younger and higher competition level participants suggest a correspondent higher involvement into the student-athlete role. However, BIMS-IT represents a distinct model with respect to the original American BIMS, determining the need of further research on the student-athletes’ identity to better clarify any socio-cultural contest effects.

## Introduction

Intensive training and competition levels create several challenges for youth elite athletes when it comes to combining their sport and educational qualifications [1, 2]. In fact, youth athletes usually start competing around 8 years of age and need of a 10-year experience to achieve elite performance, with additional 5–10 years to compete at the highest level [3]. At the same time, students spend around 30 hours per week to achieve an adequate academic career [4]. Therefore, youth elite athletes encounter several difficulties in combining both sport and educational commitments [1, 2].

In the United States, student-athletes benefit from the strict relationship between American sports and academic systems. In fact, sports are embedded within the educational environment (i.e., sports staff, training programmes, sports facilities), and athletes study and compete within the same institutions. In this framework, on a national level the American university sports represent a substantial volume of the income produced by the sports related market, with high revenue sports attracting a number of practitioners striving to pursue a professional athletic career. Therefore, American universities are strongly interested in recruiting high profile athletes as student-athletes [5]. However, some studies reported that American student-athletes frequently struggle to meet the requirements for the maintenance of the academic eligibility, being enrolled in academic institutions mainly due to their athletic profile rather than their academic capabilities [6, 7]. Thus, to ensure student-athletes’ rights to undertake a high quality educational path, the National Collegiate Athletic Association supervises university sports and student-athletes academic progresses.

Conversely, European sports are mainly structured at a club level, with no or limited relationship with the educational system. For this reason, European talented athletes may risk their academic/vocational development and/or postpone (i.e., > 24 years of age) the achievement of a degree when focused on athletic tasks. Conversely, those focused on preparing for future job opportunities may tend to dropout from sports in order to prioritize their educational path [8, 9]. Therefore, in the last decade the European Parliament [10] and the European Commission [11] identified the “dual career” (i.e., the combination of sport and education) of elite and talented athletes as an action to support their academic/vocational development and to facilitate their transition from sport into the labour market. In particular, the European Guidelines on Dual Career of Athletes [12] and the Call for Tender on the “Study on the minimum quality requirements for dual career services” [13] substantiated the European Union’s efforts in providing support to European student-athletes, encouraging the cooperation between different stakeholders, promoting best practices, and monitoring the effectiveness of dual career programmes across Europe [14, 15]. However, the European context comprises a variety of policy approaches toward the dual career [16, 17, 18], with some countries being highly supportive in ensuring opportunities for an effective combination of sport and studies for their student-athletes, whereas others are reluctant to establish and/or improve their dual career policies. In this framework, during the last decade dual career formal arrangements were at an infancy stage in Italy, with only few universities active at national and/or international levels to meet the student-athletes’ needs [18].

In the sport psychology field, the “athletic identity” encompasses psychological, emotional, and behavioural components of an athlete’s self-identity [19] and has been defined as “the degree to which an individual identifies with the athlete role”. To establish the individual’s athletic identity in the American college context, a 10-item questionnaire (Athletic Identity Measurement Scale, AIMS) was validated [20, 21]. More recently, Harrison et al. [19] structured a new measurement tool named the “Baller Identity Measurement Scale” (BIMS), which was adapted from the AIMS and also referred to the “Student Athletes’ Motivation Toward Sports and Academics Questionnaire” (SAMSAQ) [22]. This instrument was designed to assess the academic and athletic identities that might connect with motivations for performance, with a main focus on the identity constructs of the “baller” (i.e., term that resonates with the self-concept of college football and men’s basketball players competing at the highest level), which are salient with student-athletes. In a recent study [23], the BIMS was adopted to examine the impact of culture and socio-cultural contexts on the academic and athletic motivations of American male college football student-athletes. In particular, the study reported that student-athletes’ athletic and academic identities differ in relation to geography, cultural context, university attitudes toward sport/academic progresses, competition level (i.e., Division I FBS football compared with Division III women’s basketball), and media coverage. Conversely, Beamon [24] qualitatively investigated the athletic identity of African-American male former Division I student-athletes, highlighting that they are mainly characterized by both a self-identity (i.e., the degree to which an individual identifies with the athlete role) and a social identity (i.e., athletic identity related to the point of view of others), or by only self-identity [24]. To note, the latter two studies seem to provide a divergent consideration of the “baller”. In fact, Harrison et al. [23] assumed that these student-athletes’ identity encompasses both self and social traits, revealed by their ability to promote vocational and occupational areas (such as education, business, and workforce identities), and develop and implement athletic programmes and academic services for student-athletes. Conversely, Beamon [23] reported that these athletes present only a strong athletic identity, which negatively affects their career transitions especially during the retirement period, determining difficulties in redefining their identity.

In the last decade, the European scientific community was productively focused on different aspects of the student-athletes’ dual career [25] such as motivations [26, 27, 28], evaluation of dual career programmes [29, 30], polices related to the aspects of the sport and educational environments [17, 16], issues and challenges of a dual career path [4, 31], and athletic development practices [32].

However, only one study focused on the student-athletes’ identity [33], examining adolescent student-athletes’ dual career experiences (including sport, studies, and private life) during their first year at Swedish national elite sport schools. In particular, the AIMS was applied to explore how participants balanced their student and athlete roles and identities. Findings highlighted that Swedish student-athletes prioritized the sport identity with respect to the school one, with the involvement in professional sports (i.e., golf), amplifying the focus on sport commitments rather than the academic ones. Conversely, the opposite trend emerged for athletes competing in sports presenting a lower professionalization (i.e., volleyball). However, regardless of the type of sport, comparisons between the interviewees' reflections on the identity issues reported several intra-individual changes over the school year, often characterized by a transfer of prioritization from sport to school due to the recognition of the need to take care of their educational path (i.e., “you cannot live off sport, therefore you need education”).

In considering the different American and European perspectives highlighted in literature [19, 33], there is a need to provide a valid and reliable quantitative approach to evaluate the identity aspects of European university student-athletes. Thus, the present study aimed: (a) to validate the Italian version of the BIMS (i.e., BIMS-IT) to study the identity aspects of the Italian student-athletes and (b) to verify whether the identity aspects of the Italian student-athletes differ in terms of age (i.e., ≤ 24 years old, > 24 years old), gender (i.e., female, male), type of discipline (i.e., individual, team, and disciplines which can be performed both as individual and team, after reported as individual/team sport), and competition level (i.e., national sub-elite, national elite, international). It was hypothesized that the BIMS-IT: i) represents a valuable tool for the measurement of the Italian student-athletes identity (i.e., satisfactory indexes of the Exploratory Factor Analysis, EFA, and Confirmatory Factor Analysis, CFA); ii) presents a factor structure able to group items differently with respect to the original American version of the BIMS; and iii) highlights differences in identity in relation to age, gender, type of discipline, and competition level.

## Methods

### Instrumentation and Procedures

The Bioethics Committee of the University of Torino (Turin, Italy) approved this study. To adapt the original American version of the BIMS to the Italian context (i.e., BIMS-IT), the back translation method was used [34]. Prior to the translation procedures, to ascertain that the “student-athlete identity” was the main focus of the Italian version of the instrument, the terms “baller/ballers” (items 1, 2, 3, 5, 7), “ballin” (item 4, 9), “play ball” (items 6, 10), and “I don’t ball out” (item 8) were rephrased into “student-athlete/s”, “being a student-athlete”, “I’m/to be a student-athlete”, and “I’m not a student-athlete”, respectively. Then, two bilingual translators (i.e., English native language teachers in Italian universities for at least 10 years) and a monolingual English reviewer were involved in a blind translation procedure. Finally, to verify whether the Italian version of the BIMS was suitable for Italian student-athletes, a pre-test was performed administering the instrument to a representative sample (n = 12; age 21 ± 4 years), which was subsequently interviewed to ascertain reasons behind responses and to highlight problems, if any, with items’ comprehension. Thus, BIMS-IT was considered appropriate to be administered to Italian student-athletes.

Potential participants were student-athletes attending a university course at the University of Turin (Italy). They were contacted by e-mail providing information regarding the aim of the study, the anonymous nature of the responses, the absence of compensation or a tangible incentive for their participation to the study, the possibility for subjects to interrupt their participation at any time, and the link where to fill in the questionnaire as an electronic survey. Therefore, participants fully agreed to take part to the study only after submitting their responses to the entire questionnaire at the end of the web procedure.

According to the original BIMS [19], participants individually completed the 10-item BIMS-IT, indicating their level of agreement (i.e., from a minimum of 1—strongly disagree, to a maximum of 5—strongly agree) with the statements. General information (i.e., gender, age, type of discipline, competition level) was also collected at the beginning of the survey.

### Participants

To participate in the study, the following inclusion criteria for student-athletes were considered: 1) being enrolled in a University course at the University of Torino; and 2) currently competing at a National sub-elite (i.e., from local to national competition steps within the same season) or elite (i.e., from national to national or international competition steps within the same season), or International level.

### Data Analysis

To verify the applicability of the four-factor model of the American BIMS [19] for Italian university student-athletes (i.e., BIMS.IT), an Exploratory Factor Analysis (i.e., EFA; Principal Component Extraction; Varimax Rotation with Kaiser’s normalization) was performed in two main stages: i) the initial testing of the proposed four-factor model of the American version; ii) in case the four-factor model was not confirmed, the testing of different solutions (i.e., three-factor, two-factor) was planned. Coherently to the literature [35], to conduct the EFA the following criteria were adopted: i) if an item loaded on a single factor, only values ≥0.40 were taken into account; and ii) if an item loaded on two factors, a 0.32 threshold of acceptability was set for both values.

To evaluate the internal consistency of items of each BIMS-IT subscale, reliability estimates (Cronbach’s alpha coefficients) were computed, considering a Cronbach’s alpha coefficient ≥0.7 acceptable for internal consistency [36]. Furthermore, a subject to item ratio ≥10:1 was considered appropriate for EFA interpretation [35].

To verify the accuracy of the factor structure, a Confirmatory Factor Analysis (i.e., CFA; Maximum-Likelihood) was performed, using the following eight fit indexes [37]: chi-square (χ^2^), chi-square ratio (χ^2^/df), Comparative Fit Index (CFI), Goodness of Fit Index (GFI), Normed Fit Index (NFI), Tucker-Lewis Index (TLI), Root Mean Square Error of Approximation (RMSEA) and P of CLOSE fit (PCLOSE). Cut-off values for good fit were considered: ≤ 0.05 for RMSEA with not significant PCLOSE (p>0.05), ≥ 0.95 for incremental indices (CFI, NFI and TLI), ≥ 0.91for GFI, ≥ 2 for chi-square ratio.

Gender (i.e., female; male), age (i.e., ≤ 24 years; > 24 years), type of discipline (i.e., individual sport; team sport; individual/team sport), and competition levels (i.e., national sub-elite; national elite; international) were considered as independent variables to provide a detailed scenario of the Italian university student-athletes’ identity.

Items loading on two factors were used in computing composite scores for both factors, in line to previous studies focused on questionnaires for defining student-athletes’ motivation [22, 28]. In order to maintain discrete data within the entire statistical analysis, in line to the BIMS-IT scoring structure, all data related to the observed variables (i.e., gender, age, type of sport, competition level) were classified according to the sum of the identity scores (i.e., SIS) related to each BIM-IT factor, and to the percentage values based on the following formula:

SIS% = SIS X 100 / number of items related to the corresponding BIMS-IT factor X 5 (i.e., the highest possible score within the BIMS-IT scale). Differences (*p* ≤ 0.05) between the SIS values related to each factor appertaining to student-athletes of different gender, age, types of discipline, and competition levels categories were calculated by means of separate Kruskal-Wallis tests. Successively, in case of differences in relation to independent variables consisting of more than two subgroups (i.e., type of sport, year of attendance), separate Mann-Whitney U tests were performed. Then, to provide meaningful analysis for comparisons from small groups, the *phi* effect sizes (ES) between groups were also calculated, considering 0.1, 0.3, 0.5 as small, medium, and large effect sizes, respectively [38].

Statistical analyses were conducted using SPSS (21.0; SPSS, Inc., Chicago, IL) and AMOS^TM^ 21.0.

## Results

Seven hundred and sixty (23±4 yrs, range: 18–51 yrs) Italian student-athletes met the inclusion criteria and volunteered for the study (Table 1).

**Table 1:** Demographic characteristics of the Italian university student-athletes participating in the study.

Variables		*n*	%
Participants		760	100
Gender	Female	385	51
	Male	375	49
Age	≤ 24 years	616	81
	> 24 years	144	19
Type of sport	Individual	321	42
	Team	291	38
	Individual and team	148	20
Competition level	National sub-elite	511	67
	National elite	134	18
	International	115	15

Participants were mainly ≤ 24 years of age (81%) and competed in individual sports (42%). The occurrences of female (51%) and male (49%) were substantially balanced. In terms of competition level, a large proportion of student-athletes reported competing at National sub-elite level (67%).

For the Italian university student-athletes, EFA did not confirm a four-factor model which previously considered for the original American BIMS [19, 23]. Conversely, the two-factor solution resulted the most appropriate for the considered sample (i.e., explained variance = 48%; subject to item ratio = 76.0). In particular, five items and six items loaded for “Factor one” and “Factor two”, respectively, with acceptable Cronbach’s alpha coefficients for both subscales (“Factor one” = 0.84; and “Factor two” = 0.83). However, one item (#3) was removed due to low threshold of acceptability (Table 2). In general, CFA indices were found satisfactory with respect to the cut-off criteria (GFI = 0.99, NFI = 0.989, TLI = 0.987, CFI = 0.984, RMSEA = 0.036, PCLOSE = 0.886), with a significant chi-square (31.965; *p* = 0.001), and a 1.99 ratio between the hypothesized model and the sample data.

**Table 2:** Exploratory Factor Analysis and reliability estimates of the Italian version of the Baller Identity Measurement Scale (BIMS-IT) relatively to “Factor one” and “Factor two”.

#Item	“Factor one”	“Factor two”
#1. I consider myself a student-athlete.	0.81	
#2. I have many goals related to being a student-athlete.	0.79	
#3. Most of my friends are student-athletes.		
#4. Being a student-athlete is the most important part of my life.	0.56	0.60
#5. I spend more time thinking about being a student-athlete than anything else.		0.71
#6. When I’m a student-athlete, I feel good about myself.	0.63	
#7. Other people see me mainly as a student-athlete.	0.53	0.41
#8. I feel bad about myself when I do poorly when I’m not a student-athlete.		0.59
#9. Being a student-athlete is the only important thing in my life.		0.79
#10. I would be very depressed if I were injured and could not be a student-athlete.		0.51
**Alpha**	**0.84**	**0.83**

For the two BIMS-IT factors, Table 3 shows mean and standard deviation of the SIS scores, and effects between subgroups, for each of the two factors, in relation to gender, age, type of discipline, and competition level. In particular, age (*p* < 0.001) and competition level (*p* < 0.001) reported differences for both factors. In terms of age, younger (≤ 24 years old) student-athletes reported a higher identity scores related to factors (“Factor one” = 19±4, “Factor two” = 17±5) with respect to the older counterparts (> 24 years old: “Factor one” = 17±5, mean score difference expressed according to the percentage of score range, MSD% = 8%, *p* < 0.001, ES = 0.4; “Factor two” = 15±6, MSD% = 7%, *p* < 0.001, ES = 0.4). For competition level, student-athletes participating in national (“Factor one” = 21±3, MSD% = 8%, *p* < 0.001, ES = 0.4; “Factor two” = 19±5, MSD% = 12%, *p* < 0.001, ES = 0.4) and international (“Factor one” = 20±4, MSD% = 10%, *p* < 0.001, ES = 0.3; “Factor two” = 19±5, MSD% = 10%, *p* < 0.001, ES = 0.4) competitions showed higher identity scores than those practicing sport at sub-elite level.

**Table 3:** Mean and standard deviation of the sum of the identity scores (i.e., SIS), also expressed according to the percentage of score range (i.e., SIS%), with related significances, for each factor of the Italian version of the Baller Identity Measurement Scale (i.e., BIMS-IT), in relation to variables (i.e., gender, age, type of sport, competition level).

Variable		“Factor one” SIS (SIS%)	“Factor two” SIS (SIS%)
Age	≤ 24 yrs (n = 616)	19±4 (76±16%)*	17±5 (57±17%)*
	> 24 yrs (n = 144)	17±5 (68±20%)	15±6 (50±20%)
Gender	Female (n = 385)	19±5 (76±20%)	17±5 (57±17%)
	Male (n = 375)	19±4 (76±16%)	17±5 (57±17%)
Type of Sport	Individual (n = 321)	18±5 (72±20%)	17±5 (57±17%)
	Team (n = 291)	19±4 (76±16%)	17±5 (57±17%)
	Individual and team (n = 148)	19±4 (76±16%)	17±5 (57±17%)
Competition level	National sub-elite (n = 511)	18±4 (72±16%)	16±5 (53±17%)
	National elite (n = 134)	21±3 (80±12%)^¥^	19±5 (63±17%)^¥^
	International (n = 115)	20±4 (84±16%)^¥^	19±5 (63±17%)^¥^

*Different (*p* < 0.001) from “> 24 yrs” value of the same factor

^¥^ Different (*p* < 0.001) from “National sub-elite” value of the same factor.

## Discussion

This study represents the first contribution to examine the dual-career identity of Italian student-athletes and a starting point for future research in this area, in line with the recommendations of the European Union Guidelines on Dual-careers [12]. In general, findings confirmed that the BIMS-IT is a valuable tool to investigate the student-athlete identity (hypothesis 1). In fact, differently from previous studies [27, 28] which suffered of low subject to item ratios and related risks of item misclassification [35], the present study can show a large participation of student-athletes which determined a subject to item ratio of 76.0, an optimal fit of the CFA thresholds, and a high trustworthiness of results. Although the Italian sport and educational systems are governed by central bodies such as Italian Sport Federations and Italian Ministry of University and Education, respectively, in the present study, the involvement of student-athletes enrolled in a unique Italian University (i.e., University of Torino) could represent a limitation to refer findings to the general Italian contests. However, future cross-national studies including other European student-athletes are highly recommended to substantiate the psychometric properties of the BIMS-IT.

Although BIMS-IT demonstrated to be a valuable psychometric tool to investigate the identity aspects of Italian university student-athletes, a distinct model with respect to the American one [19, 23] emerged, confirming also the second hypothesis. Similarly, the European version of the Student-Athletes’ Motivation toward Sports and Academics Questionnaire (i.e., SAMSAQ-EU) [28] demonstrated to be characterized by a different factor model with respect to the original one (i.e., Student-Athletes’ Motivation toward Sports and Academics Questionnaire, SAMSAQ) [22], probably due to specific socio-cultural contexts and different relationships between the academic and sport environments. In fact, the BIMS-IT structure showed a two factors model, diverging from the four factors (i.e., social identity, exclusivity, and positive and negative affectivity) reported in the original BIMS American version [19]. In particular, in BIMS-IT, the items comprised in the “Factor one” highlight how the group membership has a profound protective effect on the sense of the self as an aspect that may satisfy the basic psychological needs of self-esteem, control, and meaning [39, 40]. Therefore, it could be termed as “Social Identity” (SI). On the other hand, the items encompassed in the “Factor two” seem to strongly refer to the perceived identity gain and loss in the context, such as high social connections and low depression, fully recognizable as student-athletes’ behaviours [39], and could be nominated as “Identity Gain (and loss)” (IG).

Regardless of the factor structure and considering the mean scores of the 9-item BIMS-IT, Italian university student-athletes reported evident divergences with respect to American collegiate-athletes that adopted the original BIMS [23]. In fact, a similarity (i.e., mean score difference expressed according to the entire BIMS-IT score range; MSD% = 2%) between the two questionnaires emerged only in correspondence of the item 10 (i.e., “I would be very depressed if I were injured and could not play Ball”). Conversely, Italian student-athletes, reported: i) higher values for the item 1 (MSD% = 50%), 2 (MSD% = 36%), 3 (MSD% = 35%), 6 (MSD% = 44%), 7 (MSD% = 28%), 8 (MSD% = 24%); and ii) lower values for item 4 (MSD% = 41%), 5 (MSD% = 26%), 9 (MSD = 1.56, MSD% = 39%); compared to American collegiate student-athletes. Therefore, similarly with respect to the student-athletes’ sport, career and academic motivations [28], these different scenarios (i.e., factor structure, and mean scores for items) strengthen the need of promoting future studies to further analyze student-athletes’ identities in relation to specific socio-cultural contexts.

The Kruskal-Wallis test applied to the Italian student-athletes’ identity factors showed effects exclusively for age and competition level, determining a partial acceptance of the third hypothesis. Both BIMS-IT factors showed that ≤ 24 years old student-athletes are characterized by a higher degree of identity with respect to the older colleagues (Factor 1, SI, MSD% = 9%; Factor 2, IG, MSD% = 7%), which probably had to postpone or prolong their academic career, suggesting that age affects the perception and pride to be a student-athlete. Although a previous study [33] was focused onto a different educational level and socio-cultural environment, it showed a modification of the student-athletes’ athletic and educational identity profile, mainly characterized by the shifted valorization from the sport area to the academic one. The higher degree of student-athletes’ identity reported by national elite (Factor 1, SI, MSD% = 8%; Factor 2, IG, MSD% = 12%) or international levels (Factor 1, SI, MSD% = 12%; Factor 2, IG, MSD% = 10%) with respect to their national sub-elite counterparts could be interpreted in favor of higher student-athletes’ difficulties in combining their sport and education commitments. In fact, the efforts required for training (i.e., occurrences of training sessions, rest between training sessions, physical intensity level, etc.) and competitions (i.e., occurrences and importance of tournaments and championships, time and efforts of travel to get competition places, etc.) are expected to be more challenging for top level student-athletes compared to those mainly competing at the local level. Therefore, greater difficulties to face, waivers, sacrifices, and extraordinary studying and sport efforts, as well as the engagement into professional sports, could have determined higher SI and IG scores for national and international student-athletes, which may shape the individual sense of self like student and athlete. Conversely, lower athletic demands seem to determine a lower feeling of belonging to the student-athlete role in the national sub-elite subgroup. This result is in line with previous literature [33], which reported that student-athletes involved in professional sports showed a priority of the sport identity with respect to the academic one.

Although limited opportunities to pursue professional athletic careers for female student-athletes were highlighted in previous studies [25, 41, 42, 43, 44], the lack of gender effects on the student-athletes’ identity is in line with the findings emerged in relation to the student-athletes’ motivational profile [28], suggesting a general development of women’s sport in Italy that could minimize this effect. Nonetheless, the gender issue represents a complex matter that should be considered when investigating student-athletes’ behavioural patterns.

In a previous study [27], Italian team sports student-athletes showed a higher academic motivation with respect to their individual sports counterparts. In particular, it was hypothesized that the preponderant focus of the Italian Military Sports Organisations on individual sports could determine a lower academic motivation in those student-athletes mainly focused on pursuing a future military career at the end of their athletic career [27]. Although motivations and identity could be easily associated in the individual profile, no effect in relation to the type of sport emerged in the present study, suggesting that the academic motivation determines different outcomes with respect to that of the student-athlete identity.

## Conclusions

The present study highlighted that the BIMS-IT is a reliable tool, which could improve the comprehension and knowledge of the student-athletes’ behavioral patterns. In addition, the quantitative approach provided in this study highlighted that the Italian student-athletes’ identity is more pronounced in the ≤ 24 years old age category, and in national elite and international competition level categories, indicating a high and effective academic and athletic involvement. However, further research focused on the student-athletes’ identity in other European Member States presenting different policies in supporting the student-athlete role [13, 18] should be envisioned, especially to better interpret the real difficulties of those student-athletes living in countries with no dual career formal arrangements [16, 17, 28], and to contribute to the new European initiatives (European Erasmus+ programme 2014–2020) in the field of sport and higher education.

## Supporting Information

S1 Dataset Questionnaire data. (XLSX)

## References

[1] Conzelmann A, Nagel S. Professional careers of the German Olympic athletes. Int Rev Sociol Sport. 2003;38:259–80.

[2] Capranica L, Millard-Stafford ML. Youth sport specialization: how to manage competition and training? Int J Sport Physiol Perform. 2011;6:572–9. 10.1123/ijspp.6.4.572 22174125

[3] Wylleman P, Reints A. A lifespan perspective on the career of talented and elite athletes: perspectives on high-intensity sports. Scand J Med Sci Sports. 2010;2:88–94. 10.1111/j.1600-0838.2010.01194.x 20840566

[4] Aquilina D. A study of the relationship between elite athletes' educational development and sporting performance. Int J Hist Sport. 2013;30:374–92.

[5] Gatmen EJP. Academic exploitation: the adverse impact of college athletics on the educational success of minority student-athletes. Seattle J. Soc. Just. 2012;10:509–83.

[6] Simons HD, Van Rheenen D, Covington MV. Academic motivation and the student athlete. J Coll Stud Dev. 1999;40:151–62.

[7] Aries E, McCarthy D, Salovey P, Banaji MR. A Comparison of Athletes and Non-athletes at Highly Selective Colleges: Academic Performance and Personal Development. Res High Educ. 2004;45:577–602.

[8] Amara M, Aquilina D, Henry I. Education of young sportspersons (lot 1). Report Finale, Directorate-General Education and Culture, Brussels 2004. Available from: http://ec.europa.eu/sport/library/documents/c3/pmp-study-dual-career_en.pdf.

[9] Istituto Nazionale di Statistica-ISTAT. La pratica sportiva in Italia. 2007. Available from: http://www3.istat.it/salastampa/comunicati/non_calendario/20070620_00.

[10] European Parliament. Combining sport and education. Support for athletes in the EU 2003.

[11] European Commission. Commission staff document: action plan ‘Pierre de Coubertin’, accompanying document to the white paper on sport. Directorate-General Education and Culture, Brussels 2007. Available from: http://eur-lex.europa.eu/legal-content/EN/TXT/?uri=CELEX%3A52007SC0934.

[12] European Commission. Guidelines on Dual-careers of Athletes Recommended Policy Actions in Support of Dual-careers in High-Performance Sport 2012. Available from: http://ec.europa.eu/sport/news/20130123-eu-guidelines-dualcareers_en.htm.

[13] Amsterdam University of Applied Science, Birch Consultants, the Talented Athlete Scholarship Scheme, the Vrije Universiteit Brussel, and European Student as Athlete network. Study on the minimum quality requirements for dual career services. Executive summary 2016. Avaiable from: http://bookshop.europa.eu/en/study-on-the-minimum-quality-requirements-for-dual-career-services-pbNC0216013/.

[14] European Commission. Resolution of the Council and of the Representatives of the Governments of the Member States, meeting within the Council, of 21 May 2014 on the European Union Work Plan for Sport (2014–2017) 2014. Available from: http://eur-lex.europa.eu/legal-content/EN/TXT/PDF/uri=CELEX:42014Y0614(03)andfrom=EN.

[15] European Commission. Resolution of the Council and of the Representatives of the Governments of the Member States, meeting within the Council, of 21 May 2014 on the European Union Work Plan for Sport (2014–2017) 2014. Available from: http://eur-lex.europa.eu/legal-content/EN/TXT/PDF/?uri=CELEX:42014Y0614(03)andfrom=EN.

[16] Aquilina D, Henry I. Elite athletes and university education in Europe: a review of policy and practice in higher education in the European Union Member States. Int J of Sport Policy. 2010;1:25–47.

[17] Henry I. Athlete Development, Athlete Rights and Athlete Welfare: A European Union Perspective. IJHS. 2013;30:356–73.

[18] Capranica L, Guidotti F. Research for cult committee qualifications/dual careers in sports. European Parliament: Directorate-General for internal policies. Policy Department. Structural and cohesion policies: Cultural and Education 2016. Available from: http://www.europarl.europa.eu/RegData/etudes/STUD/2016/573416/IPOL_STU(2016)573416_EN.pdf.

[19] Harrison CK, Rasmussen J, Connolly CM, Janson NK, Bukstein S, Parks C. The Need for the Baller Identity Measurement Scale (BIMS) in Assessing Academic and Athletic Identities in Society. J Stud Sports Athl Educ. 2010;4(3): 325–32.

[20] Brewer BW, Van Raalte JL, Linder DE. Athletic Identity: Hercules’ muscles or Achilles heel? Int J Sport Psychol. 1993;24:237–54.

[21] Brewer BW, Cornelius AE. Norms and factorial invariance of the Athletic Identity Measurement Scale. Academic Athletic Journal. 2001;15:103–13.

[22] Gaston-Gayles JL. The factor structure and reliability of the Student Athletes Motivation toward Sports and Academics Questionnaire (SAMSAQ). J Coll Student Dev. 2005;46:317–27.

[23] Harrison CK, Tranyowicz L, Bukstein S, McPherson-Botts G, Lawrence SM. I am what I am? The Baller Identity Measurement Scale (BIMS) with a Division I football team in American higher education. Sport Sci Health. 2014;10:53–8.

[24] Beamon K. “I’m a Baller”: athletic identity foreclosure among African-American former student-athletes. J Afr Am Stud. 2012;16:195–208.

[25] Guidotti F, Capranica L. Management sportivo femminile e carriera universitaria nelle scienze motorie: la condizione attuale, le opinioni delle manager e delle docenti universitarie e le nuove proposte (Female sport management and academic career in sport sciences: Present condition, women sport managers’ and university professors’ opinions and perspectives). Rivista Trimestrale di Scienza dell’Amministrazione. 2013;1:85–104.

[26] Guidotti F, Minganti C, Cortis C, Piacentini MF, Tessitore A, Capranica L. Validation of the Italian Version of the Student Athletes Motivation Toward Sport and Academics Questionnaire. Sport Sci Health. 2013;9,51–8.

[27] Lupo C, Tessitore A, Capranica L, Rauter S, Doupona Topic M. Motivation for a dual-career: Italian and Slovenian student-athletes. Kin Si. 2012;18:47–56.

[28] Lupo C, Guidotti F, Goncalves CE, Moreira L, Doupona Topic M, Bellardini H, et al Motivation Toward Dual-Career of European Student-Athletes. Eur J Sport Sci. 2015;15:151–60. 10.1080/17461391.2014.940557 25145585

[29] van Rens FECA, Elling A, Reijgersberg N. Topsport Talent Schools in the Netherlands: A retrospective analysis of the effect on performance in sport and education. Int Rev Sociol Sport; 2015;50(1):64–82.

[30] McCormack C, Walseth K. Combining elite women’s soccer and education: Norway and the NCAA. Soccer Soc. 2013;14(6):887–97.

[31] Ryba TV, Stambulova NB, Ronkainen NJ, Bundgaard J, Selänne H. Dual career pathways of transnational athletes. Psychol Sport Exerc. 2015;21:121–34.

[32] Henriksen K, Larsen CH, Christensen MK. Looking at success from its opposite pole: The case of a talent development golf environment in Denmark. Int J Sport Exerc Psychol. 2014;12(2):134–49.

[33] Stambulova NB, Engström C, Francks A, Linnér L, Lindhal K. Searching for an optimal balance: Dual career experiences of Swedish adolescent athletes. Psychol Sport Exerc. 2015;21:4–14.

[34] Su CT, Parham LD. Case Report—Generating a valid questionnaire translation for cross-cultural use. Am J Occup Ther. 2002;56:581–5. 12269514 10.5014/ajot.56.5.581

[35] Costello AB, Osborne JW. Exploratory factor analysis: Four recommendations for getting the most from your analysis. Pract Assess Res Eval. 2005;10:1–9.

[36] O’Donoghue P. Statistics for sport and exercise studies: An introduction. Abingdon, Routledge; 2012.

[37] Jackson DL, Gillaspy JA Jr, Purc-Stephenson R. Reporting practices in confirmatory factor analysis: an overview and some recommendations. Psychol. Methods. 2009;14:6–23. 10.1037/a0014694 19271845

[38] Huck SW. Reading statistics and research (3rd ed). New York, NY: Addition, Wesly Longman; 2000 p. 28–629.

[39] Armstrong S, Oomen-Early J. Social Connectedness, Self-Esteem, and Depression Symptomatology Among Collegiate Athletes Versus Nonathletes. J Am Coll Health. 2009;57(5):521–5. 10.3200/JACH.57.5.521-526 19254893

[40] Cruwys T, Greenaway KH, Haslam SA. The stress of passing through an educational bottleneck: A longitudinal study of psychology honours students. Aust Psychol. 2015;50(5):372–81.

[41] International Olympic Committee. Women and sport progress report: A review of the IOC policy and activities to promote women in and through sport. Third World Conference on Women and Sport; 2004; Marrakech, Morocco.

[42] International Olympic Committee. The Los Angeles Declaration. 2012. Available from: http://www.olympic.org/Documents/Commissions_PDFfiles/women_and_sport/Los-Angeles-Declaration-2012.pdf.

[43] International Working Group on Women and Sport. The Sydney scoreboard. 2012. Available from: http://www.sydneyscoreboard.com/.

[44] Pfister G. Women in sport–gender relations and future perspectives. Sport in Society. 2010;13:234–248.

